# Associations between model-predicted rivaroxaban exposure and patient characteristics and efficacy and safety outcomes in the prevention of venous thromboembolism

**DOI:** 10.1007/s11239-020-02078-8

**Published:** 2020-04-23

**Authors:** Isabel Reinecke, Alexander Solms, Stefan Willmann, Theodore E. Spiro, Gary Peters, Jeffrey I. Weitz, Wolfgang Mueck, Dirk Garmann, Stephan Schmidt, Liping Zhang, Keith A. A. Fox, Scott D. Berkowitz

**Affiliations:** 1 Bayer AB, Solna, Sweden; 2 Clinical Pharmacometrics, Bayer AG, Berlin, Germany; 3 Clinical Pharmacometrics, Bayer AG, Wuppertal, Germany; 4 Bayer U.S., LLC, Research & Development, Pharmaceuticals, 100 Bayer Boulevard, Whippany, NJ 07981 USA; 5 Janssen Research & Development, LLC, Raritan, NJ USA; 6 McMaster University, and the Thrombosis & Atherosclerosis Research Institute, Hamilton, ON Canada; 7 Clinical Pharmacokinetics, Bayer AG, Wuppertal, Germany; 8 Center for Pharmacometrics and Systems Pharmacology, Department of Pharmaceutics, College of Pharmacy, University of Florida, Orlando, FL USA; 9 Centre for Cardiovascular Science, The University of Edinburgh, Edinburgh, UK

**Keywords:** Drug monitoring, Exposure–response, Rivaroxaban, Venous thromboembolism

## Abstract

Anticoagulant plasma concentrations and patient characteristics might affect the benefit–risk balance of therapy. The study objective was to assess the impact of model-predicted rivaroxaban exposure and patient characteristics on outcomes in patients receiving rivaroxaban for venous thromboembolism (VTE) prophylaxis (VTE-P) after hip/knee replacement surgery. Post hoc exposure–response analyses were conducted using data from the phase 3 RECORD1–4 studies, in which 12,729 patients were randomized to rivaroxaban 10 mg once daily or enoxaparin for ≤ 39 days. Multivariate regression approaches were used to correlate model-predicted individual rivaroxaban exposures and patient characteristics with outcomes. In the absence of measured rivaroxaban exposure, exposure estimates were predicted based on individual increases in prothrombin time (PT) and by making use of the known correlation between rivaroxaban plasma concentration and dynamics of PT. No significant associations between rivaroxaban exposure and total VTE or major bleeding were identified. A significant association between exposure and a composite of major or non-major clinically relevant (NMCR) bleeding from day 4 after surgery was observed. The relationship was shallow, with an approximate predicted absolute increase in a composite of major or NMCR bleeding from 1.08 [95% confidence interval (CI) 0.76–1.54] to 2.18% (95% CI 1.51–3.17) at the 5th and 95th percentiles of trough plasma concentration, respectively. In conclusion, based on the underlying data and analysis, no reliable target window for exposure with improved benefit–risk could be identified within the investigated exposure range. Hence, monitoring rivaroxaban levels is unlikely to be beneficial in VTE-P.

**Electronic supplementary material:**

The online version of this article (10.1007/s11239-020-02078-8) contains supplementary material, which is available to authorized users.

## Highlights

- Employing multivariate regression approaches, post hoc exposure–response analyses were performed using data from the RECORD1–4 studies to assess the impact of predicted rivaroxaban exposure and patient characteristics on clinical outcomes.
- Rivaroxaban exposure–response relationships with both efficacy and safety were shallow sloped or absent within the investigated exposure range.
- Monitoring rivaroxaban levels is unlikely to be beneficial when used for thromboprophylaxis.

## Introduction

Rivaroxaban, an oral direct factor Xa inhibitor, is approved for several indications, including venous thromboembolism prophylaxis (VTE-P) in adults undergoing elective total hip/knee replacement (THR/TKR) surgery [1].

Rivaroxaban was developed to provide predictable anticoagulation with fixed-dose administration, without the need to routinely measure drug levels or perform coagulation assays for dose adjustment. This approach is supported by the high bioavailability of rivaroxaban when administered with food, and the low potential for food and drug interactions [2–4], which minimize variability in rivaroxaban exposure [3, 4].

Data from phase 2 dose-ranging studies in patients receiving rivaroxaban for VTE-P after THR (ODIXa-Hip2, ODIXa-Hip-OD) or TKR (ODIXa-Knee) [5–8] supported the investigation of rivaroxaban 10 mg once daily (OD) in the phase 3 RECORD program [9–14], leading to approval of this regimen for VTE-P [1]. Phase 2 data were also used to construct population pharmacokinetic (popPK) models to characterize the pharmacokinetics of rivaroxaban in patients undergoing THR/TKR [15, 16]. The models reliably predicted the pharmacokinetic profile of rivaroxaban and demonstrated a strong correlation between prothrombin time (PT) prolongation (obtained using a sensitive thromboplastin reagent) and rivaroxaban plasma concentration [2, 15, 16].

Patient characteristics affect the benefits and risks (e.g., bleeding) associated with anticoagulation therapy [1, 17]; for example, advanced age and impaired renal function are associated with increased rivaroxaban exposure [1]. In patients receiving anticoagulants for VTE-P, factors such as previous history of venous thromboembolism (VTE), active cancer and its treatments, and surgery type, if applicable, affect the risk of VTE and/or bleeding [18–21].

Given that rivaroxaban exposure may vary between patients, it has been proposed that therapeutic drug monitoring (i.e., plasma-concentration-based dose adjustment) may enhance the individual benefit–risk ratio of treatment [22]. Such treatment individualization requires a robust understanding and quantification of the association between exposure and safety and efficacy. Owing to a lack of observed pharmacokinetic data in the phase 3 RECORD trials, individual rivaroxaban exposure was predicted using a previously developed popPK model, individual covariates and PT measurements [23, 24]. Using data from the RECORD1–4 studies and individually predicted derived rivaroxaban exposure parameters, post hoc model-predicted exposure–response analyses were performed to assess the impact of rivaroxaban exposure and patient characteristics on clinical outcomes in patients receiving rivaroxaban for VTE-P after elective THR surgery (35 days of treatment) or TKR surgery (12 days of treatment). The data reported here accompany the results of a similar analysis in which the impact of rivaroxaban exposure and patient characteristics on clinical outcomes were assessed in patients receiving rivaroxaban for VTE treatment.

## Methods

### Study design

Full details of the methodology and ethical conduct of RECORD1–4 (Table 1) have been published previously [9, 10, 13, 14]. Briefly, in RECORD1–4, 12,729 patients who were undergoing elective THR or TKR were randomized to receive oral rivaroxaban (10 mg once daily [OD]) or subcutaneous enoxaparin for ≤ 39 days (THR) or ≤ 14 days (TKR). The efficacy outcome evaluated in the current exposure–response analysis was total VTE (i.e., any objectively documented symptomatic or asymptomatic deep vein thrombosis [DVT; proximal and/or distal], non-fatal pulmonary embolism [PE] or death). Safety outcomes included major bleeding and the composite outcome of major or non-major clinically relevant (NMCR) bleeding (Table 1). The exposure–efficacy analysis included asymptomatic events detected by bilateral venography and objectively confirmed symptomatic events from the first day of rivaroxaban administration until day 42 (RECORD1&2) or day 17 (RECORD3&4). The exposure–safety analysis included events occurring from the first day of rivaroxaban administration until 2 days after the last dose. Bleeding events were separated into events occurring up to 3 days after surgery (on days 1–4) and more than 3 days after surgery (after day 4), to account for the influence of the surgical procedure on bleeding.

**Table 1:** Description of the studies and outcomes included in the exposure–response analyses

Study	RECORD1 [9]	RECORD2 [10]	RECORD3 [13]	RECORD4 [14]
Population	Patients undergoing elective THR		Patients undergoing elective TKR	
Total number of patients randomized	12,729			
Pertinent exclusion criteria	Concomitant use of HIV protease inhibitors		Concomitant use of strong CYP3A4 inhibitors, such as ketoconazole or protease inhibitors	
Rivaroxaban dose and regimen	10 mg OD 35 ± 4 days		10 mg OD 12 ± 2 days	
Comparator dose and regimen	Enoxaparin 40 mg OD 35 ± 4 days	Enoxaparin 40 mg OD 12 ± 2 days followed by placebo	Enoxaparin 40 mg OD 12 ± 2 days	Enoxaparin 30 mg BID 12 ± 2 days
Maximum follow-up	65 days			
Mean treatment duration	Rivaroxaban: 33.5 days Enoxaparin: 33.7 days		Rivaroxaban: 11.7 days Enoxaparin: 11.0 days	
Total number of patients for ER analysis	6097 (safety) 4246 (efficacy)			
Efficacy outcomes for ER analysis	Total VTE: composite of objectively documented asymptomatic or symptomatic DVT (proximal and/or distal), non-fatal PE and death from any cause			
Safety outcomes for ER analysis	1. RECORD major bleeding^a^ (days 1–4 and after day 4) 2. Major or NMCR^b^ bleeding (days 1–4 and after day 4)			

*BID* twice daily, *CYP3A4* cytochrome P450 3A4, *DVT* deep vein thrombosis, *ER* exposure–response, *HIV* human immunodeficiency virus, *NMCR* non-major clinically relevant, *OD* once daily, *PE* pulmonary embolism, *THR* total hip replacement, *TKR* total knee replacement, *VTE* venous thromboembolism

^a^RECORD major bleeding was defined as: fatal bleeding; bleeding into a critical organ; bleeding that required re-operation; or clinically overt extra-surgical-site bleeding associated with a decrease in hemoglobin of ≥ 2 g/dL or requiring a transfusion of ≥ 2 units of whole blood or packed cells

^b^NMCR bleeding was defined as: overt bleeding that did not meet the criteria for major bleeding but was associated with medical intervention; unscheduled contact with a physician; interruption or discontinuation of a study drug; or discomfort or impairment of activities of daily life

### Patient characteristics

Patient characteristics considered in the exposure–response evaluation (including potential risk factors for clinical outcomes) were identified a priori based on a literature review [20, 25–29] and experience in RECORD1–4 [9, 10, 13, 14]. Continuous variables, including age, were categorized to aid interpretation.

### Model-predicted rivaroxaban exposure

Because rivaroxaban plasma concentrations were not measured in the phase 3 RECORD studies, a previously reported integrated popPK model was employed to predict individual rivaroxaban exposure estimates using patient characteristics known to influence rivaroxaban pharmacokinetics [24]. Trough plasma concentration (C_trough_), maximum plasma concentration (C_max_) and area under the plasma concentration–time curve from 0 to 24 h (AUC_0–24_) at steady state were predicted for each patient based on individual characteristics (age, weight, renal function measured as calculated creatinine clearance [CrCl] using the Cockcroft–Gault equation, and sex) and rivaroxaban dose. Using patient characteristics alone to predict individual rivaroxaban exposure might not adequately reflect the expected variability; therefore, a new approach to enhance model-predicted rivaroxaban exposures based on the collateral correlation between rivaroxaban plasma concentration and measured PT was applied to 5293 patients receiving rivaroxaban for VTE-P, as described previously [23].

Exposure–efficacy and exposure–safety analyses were performed in patients who received at least one blinded dose of study drug, had undergone the appropriate surgery and had an adequate assessment of thromboembolism, and for those who underwent randomization and received at least one dose of study drug, respectively.

### Regression analyses

Exposure–response relationships were evaluated using logistic regression with application of penalized likelihood (Firth method) to avoid small-sample bias [30]. Time-to-event analysis was not expected to provide additional information in these contexts owing to the short (≤ 39 days) treatment duration. The analysis was conducted using R (version 3.3.0) and the logistf and survival packages.

Relationships between rivaroxaban exposure metrics, patient characteristics and each of the efficacy and safety outcomes were quantified using the following methods. Initially, univariate regression analyses were performed using C_trough_, C_max_ or AUC_0–24_ as independent variables, assuming a linear relationship for the continuous exposure measures (logistic regression). The exposure metric most strongly associated with the occurrence of an event, indicated by the lowest Akaike information criterion (AIC) value generated by the univariate analyses, was then combined with the selected patient characteristics for the VTE-P indication as independent variables for predicting the probability of the outcomes in multivariate regression analyses (the full model). Age and CrCl were expected to influence outcomes [1], and were, therefore, forced into the models regardless of their statistical significance. This forced inclusion was done to avoid bias in the variable selection process due to confounding variables, because a patient’s CrCl and age, for example, correlate with rivaroxaban exposure. An additional covariate forced into the model was type of surgery (THR or TKR) for the efficacy analyses. With selected variables forced into the model, backward elimination, based on AIC values, was performed on the other variables until no further variable was removed. All statistically non-significant variables, with the exception of the forced input variables, were removed to generate the final model. Statistical significance refers to covariates, including exposure, showing a p value no greater than 0.01 according to the likelihood ratio test.

If exposure was included in the final model, odds ratios (ORs) were generated for the variables in the final models and shown in forest plots. The reference category was the most-commonly observed category for the variable, except for region, for which Western Europe was set as the reference. For exposure metrics, the median value of each dose was set as the reference to represent the typical exposure in a patient at that dose level. The final models were used to simulate the probability of efficacy or safety events versus exposure in a typical patient population (i.e., with individual patient characteristics set to reference values).

## Results

### Patient characteristics

Supplemental Table 1 shows the patient characteristics selected for evaluation. Supplemental Table 2 shows the count and proportion of patients in the RECORD1–4 studies (safety population, n = 6097; efficacy population, n = 4246) with each characteristic. Among the 6097 patients included in the safety analysis, almost half (47%) were < 65 years of age, 38% were 65–75 years of age, 15% were > 75 years of age, 61% were female and 55% underwent THR. Overall, 7% had CrCl < 50 mL/min and 1% had active malignancy at randomization.

### Rivaroxaban exposure predictions and event rates

Rivaroxaban exposure predictions are shown in Supplemental Table 3. The derived, model-based exposure metrics showed moderate variability, with C_trough_ being the most variable parameter (coefficient of variation: 61.3–66.6%). The predicted exposure metrics were all highly correlated (correlation coefficient ≥ 0.60) within a given individual. The observed efficacy and safety outcome event rates are presented in Supplemental Table 4.

### Regression analyses

Results of the final exposure–response models are summarized in Table 2.

**Table 2:** Results of the final exposure–response models

Variables	Efficacy	Safety	
	Total VTE	Major bleeding	Major or NMCR bleeding
TKR^a^	X	NA	NA
Age^a^	n.s	n.s	n.s
CrCl^a^	n.s	n.s	n.s
Best exposure	n.s	n.s	C_trough_ (after day 4 only)
Other significant covariate	None	None	Hospital region, sex (days 1–4 only)

X denotes statistically significant exposure–response relationship (p ≤ 0.01)

*CrCl* creatinine clearance, *C*_*trough*_ trough plasma concentration, *NA* not applicable, *NMCR* non-major clinically relevant, *n.s*. not significant, *TKR* total knee replacement, *VTE* venous thromboembolism

^a^Forced input variables

### Exposure–efficacy analysis

In the univariate regression analysis, C_max_ was associated with the lowest AIC value when fitting the exposure metrics C_trough_, C_max_ or AUC_0–24_, and was therefore selected for further investigation (Supplemental Table 5). Given that model-predicted C_max_ was removed during the backward elimination process, no significant association between rivaroxaban exposure and the composite efficacy outcome of total VTE was present in the RECORD1–4 studies (Supplemental Table 6). Age and CrCl were not significantly associated with total VTE in the final model; however, patients undergoing TKR were more likely to experience total VTE than those undergoing THR (OR: 5.91 [95% confidence interval (CI) 4.09–8.76]) (Supplemental Table 7). No significant associations between other patient characteristics and total VTE were identified.

### Exposure–safety analysis

C_max_ was selected for further investigation in the exposure–safety analyses for bleeding events occurring ≤ 3 days after THR/TKR (on days 1–4), because it was the exposure metric associated with the lowest AIC value in the univariate regression analyses. For similar reasons, AUC_0–24_ and C_trough_ were selected for inclusion in the full models for major bleeding events occurring after day 4 and for a composite of major or NMCR bleeding events occurring after day 4, respectively (Supplemental Table 5).

For major bleeding during days 1–4 and after day 4, the overall number of events was low (11 of 6097 patients [0.18%] during days 1–4, and 11 of 5995 patients after day 4 [0.18%]; Supplemental Table 4) and no significant exposure–response relationship could be established (Supplemental Table 8). In addition, there was no significant association between any of the evaluated patient characteristics, including the forced input variables of age and CrCl, and major bleeding (Supplemental Table 9).

**Fig. 1:**
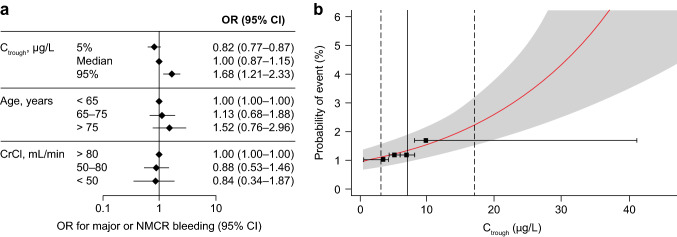
**a** ORs for the composite safety outcome of major or NMCR bleeding after day 4 in patients receiving rivaroxaban for VTE-P after elective hip or knee replacement surgery (No patient characteristics, including the forced input variables of age or CrCl, were identified as risk factors for the composite of major or NMCR bleeding after day 4. Results of the likelihood ratio test for the final safety outcome models are shown in Supplemental Table 9). **b** Probability of a major or NMCR bleeding event after day 4 versus rivaroxaban C_trough_ in this population. The solid red line represents the predicted probability, and the shaded area represents 95% CIs. Vertical dashed lines indicate the 5th and 95th percentiles of exposure in the population, and the vertical solid line indicates the median. Horizontal solid black lines represent quartiles of exposure in the reference population (age < 65 years and CrCl > 80 mL/min), and black squares represent the observed fraction of events at the median of exposure within each quartile of exposure. *CI* confidence interval, *CrCl* creatinine clearance, *C*_*trough*_ trough plasma concentration, *NMCR* non-major clinically relevant, *OR* odds ratio, *VTE-P* venous thromboembolism prophylaxis

For the composite of major or NMCR bleeding during days 1–4, no significant exposure–response relationship could be established (Supplemental Table 8). Of the patient characteristics evaluated, only male versus female sex (OR 3.05 [95% CI 1.92–4.93]; p < 0.00001) and hospital region (OR 1.63 [95% CI 0.98–2.70] for the USA/Canada vs Western Europe; p = 0.001) were significantly associated with this outcome (Supplemental Table 9). For major or NMCR bleeding after day 4, a statistically significant exposure–response relationship was observed, with ORs of 0.82 (95% CI: 0.77–0.87) and 1.68 (95% CI: 1.21–2.33) for C_trough_ at the 5th and 95th percentiles of exposure versus the median, respectively (Fig. 1a). No patient characteristics, including the forced input variables of age and CrCl, were significantly associated with the composite of major or NMCR bleeding after day 4 (Supplemental Table 9; Fig. 1a).

### Expected probability of safety events during treatment with rivaroxaban

The relationship between model-predicted exposure (C_trough_) and the probability of major or NMCR bleeding with rivaroxaban treatment after day 4 was shallow, with an approximate predicted absolute increase in a composite of major or NMCR bleeding from 1.08 (95% CI 0.76–1.54) to 2.18% (95% CI 1.51–3.17) at the 5th and 95th percentiles of C_trough_, respectively (Fig. 1b). Although a significant relationship was identified, the shape of this relationship could not be reliably evaluated, most likely due to the overall low event rates. In particular, at the upper end of the investigated exposure range the uncertainty in the model-predicted event rate was relatively high as indicated by the wide confidence interval (Fig. 1b).

## Discussion

In this analysis, no exposure–response relationships were observed for total VTE and major bleeding events in patients receiving rivaroxaban 10 mg OD for VTE-P. However, significant reductions in the risk of VTE were observed with rivaroxaban versus enoxaparin in the RECORD1–4 studies [9, 10, 13, 14]. Taken together, these findings support the rivaroxaban 10 mg OD fixed-dose regimen.

Exposure, particularly C_trough_, was the predominant risk factor for a composite of major or NMCR bleeding after THR/TKR from day 4, with a shallow-sloped exposure–response relationship. These findings are consistent with data from VTE-P phase 2 trials, in which no significant dose–response relationship was observed for total VTE, but postoperative bleeding events increased dose-dependently over the range 5–40 mg OD and 2.5–30 mg twice daily [6, 7, 31].

In this analysis, only male versus female sex and hospital region were significantly associated with major or NMCR bleeding during days 1–4 after THR/TKR. Patient characteristics, including age and CrCl, were not significantly associated with major or NMCR bleeding after day 4 or with major bleeding alone.

Consistent with our findings, a previous study in patients receiving apixaban after THR/TKR reported no statistically significant exposure–efficacy relationship [32]. Predicted bleeding frequencies for patients with risk factors for high apixaban exposure were similar to those for the reference population, supporting the recommendation that apixaban dose adjustment is not necessary to reduce bleeding risk in these patients [32]. Furthermore, since their introduction for VTE-P, other anticoagulants (unfractionated heparin, low molecular weight heparins, indirect and direct factor Xa inhibitors, and direct thrombin inhibitors) have been used in fixed doses, without adjustment.

Limitations of this analysis include the paucity of direct pharmacokinetic measurements and consequent use of model-predicted exposure data, which could not fully reproduce the inter-patient variability expected in a real-world patient population. The exposure–response analyses were post hoc, and the phase 3 studies included, which were of relatively short duration, were not designed to evaluate exposure–response relationships and the impact of patient characteristics on outcomes. Finally, these analyses were based on only the approved 10 mg OD dosing regimen for VTE-P; exposure–response relationships with other regimens cannot be excluded. To more reliably draw conclusions on the utility of a therapeutic drug monitoring treatment approach, and to establish a treatment algorithm that is trusted to improve individual patient treatment outcome, further systematic evaluation of data and methods and correlation with clinical events in outcomes trials would be needed.

## Conclusions

In this analysis, model-predicted rivaroxaban exposure–response relationships were shallow or absent for both safety and efficacy outcomes. Based on the underlying studies, no reliable target window for exposure with improved benefit–risk could be identified within the investigated exposure range and there was no evidence that the benefit–risk balance of rivaroxaban would be enhanced by implementing therapeutic drug monitoring as a routine measure [33]. These results support the approved, fixed-dose rivaroxaban regimen for VTE-P. However, as observed with other direct oral anticoagulants, evaluating patient characteristics, particularly renal function, also provides valuable information when considering treatment with rivaroxaban.

## Electronic supplementary material

Below is the link to the electronic supplementary material.

Supplementary file1 (DOCX 45 kb)
