# Supplementary material for: Associations between model-predicted rivaroxaban exposure and patient characteristics and efficacy and safety outcomes in the prevention of venous thromboembolism
Source: J Thromb Thrombolysis. 2020 Apr 23;50(1):12–9. doi: 10.1007/s11239-020-02078-8 (PMC7293976; doi:10.1007/s11239-020-02078-8)
Supplement: Supplementary file 1 — Supplementary file1 (DOCX 45 kb) [file 11239_2020_2078_MOESM1_ESM.docx]

Supplemental Appendix

**Associations between model-predicted rivaroxaban exposure and patient characteristics and efficacy and safety outcomes in the prevention of venous thromboembolism**

*Journal of Thrombosis and Thrombolysis*

Isabel Reinecke^1^ • Alexander Solms^2^ • Stefan Willmann^3^ • Theodore E. Spiro^4^ • Gary Peters^5^ • Jeffrey I. Weitz^6^ • Wolfgang Mueck^7^ • Dirk Garmann^3^ • Stephan Schmidt^8^ • Liping Zhang^5^ • Keith A. A. Fox^9^ • Scott D. Berkowitz^4^

^1^ Bayer AB, Solna, Sweden, on behalf of Bayer AG, Research & Development, Pharmaceuticals, Berlin, Germany

^2^ Clinical Pharmacometrics, Bayer AG, Berlin, Germany

^3^ Clinical Pharmacometrics, Bayer AG, Wuppertal, Germany

^4^ Bayer U.S., LLC, Research & Development, Pharmaceuticals, Whippany, NJ, USA

^5^ Janssen Research & Development, LLC, Raritan, NJ, USA

^6^ McMaster University, and the Thrombosis & Atherosclerosis Research Institute, Hamilton, ON, Canada

^7^ Clinical Pharmacokinetics, Bayer AG, Wuppertal, Germany

^8^ Center for Pharmacometrics and Systems Pharmacology, Department of Pharmaceutics, College of Pharmacy, University of Florida, Orlando, FL, USA

^9^ Centre for Cardiovascular Science, The University of Edinburgh, Edinburgh, UK

**Address for correspondence** Scott D. Berkowitz, MD, Bayer U.S., LLC, Clinical Development – Thrombosis, 100 Bayer Boulevard, Whippany, NJ 07981, USA

Tel.: +1 862 404 3485

E-mail: [scott.berkowitz@bayer.com](mailto:scott.berkowitz@bayer.com)

**Supplemental Table 1** Definition of patient characteristics for inclusion in exposure–response models

| **Covariate^a^** | **Categories^b^** | **Exposure–efficacy models** | **Exposure–safety models** |
| --- | --- | --- | --- |
| Patient characteristics included in the exposure–response models regardless of significance level | | | |
| Type of surgery | THR, TKR | 🗹 | **–** |
| Age | < 65, 65–75, > 75 years | 🗹 | 🗹 |
| Baseline renal function (CrCl) | < 50, 50–80, > 80 mL/min | 🗹 | 🗹 |
| Patient characteristics for potential inclusion in the exposure–response models | | | |
| Geographic region | Western Europe, Eastern Europe, USA/Canada, other | 🗹 | 🗹 |
| Revision surgery | Yes, no | **–** | 🗹 |
| Duration of surgery ≥ 2 hours | Yes, no | 🗹 | **–** |
| Sex | Male, female | 🗹 | 🗹 |
| Body weight | < 70, 70–90, > 90 kg | 🗹 | 🗹 |
| Body mass index | < 25, 25–< 35, ≥ 35 kg/m^2^ | 🗹 | **–** |
| Low Hb prior to surgery (< 13 g/dL for men and < 12 g/dL for women) | Yes, no | **–** | 🗹 |
| Any VTE risk factor | Yes, no | 🗹 | **–** |
| History of VTE | Yes, no | 🗹 | **–** |
| Cardiac insufficiency | Yes, no | 🗹 | **–** |
| Active malignancy | Yes, no | 🗹 | 🗹 |
| Thrombophilia | Yes, no | 🗹 | 🗹 |
| Severe varicosis | Yes, no | 🗹 | 🗹 |
| History of bleeding (per SMQ) | Yes, no | **–** | 🗹 |
| History of hypertension | Yes, no | 🗹 | 🗹 |
| History of CV disease | Yes, no | 🗹 | **–** |
| Drug use status | | | |
| PAI or ASA use while receiving rivaroxaban | Yes, no | **–** | 🗹 |
| NSAID use while receiving rivaroxaban | Yes, no | **–** | 🗹 |

*ASA* acetylsalicylic acid, *CrCl* creatinine clearance, *CV* cardiovascular, *Hb* hemoglobin, *NSAID* non-steroidal anti‑inflammatory drug, *PAI* platelet aggregation inhibitor, *SMQ* standardized MedDRA (Medical Dictionary for Regulatory Activities) query, *THR* total hip replacement, *TKR* total knee replacement, *VTE* venous thromboembolism

^a^Missing values for covariates were filled by carry forward if a previous value for a patient was unavailable or by carry backward if only a later variable was recorded. In cases where the covariate value was not recorded at any time during the study for the patient, the median value calculated from otherwise similar patients in the population dataset was used

^b^Based on current labels or clinical/statistical rationale

**Supplemental Table 2** Counts of patient characteristics for inclusion in exposure–response models

| **Covariate** | **Category** | **Number of patients** | **Percent^a^** |
| --- | --- | --- | --- |
| **Safety population (n = 6097)** | | | |
| Age | < 65 years | 2876 | 47.2 |
|  | 65–75 years | 2327 | 38.2 |
|  | > 75 years | 894 | 14.7 |
| Baseline renal function (CrCl) | > 80 mL/min | 3600 | 59.0 |
|  | 50–80 mL/min | 2098 | 34.4 |
|  | < 50 mL/min | 399 | 6.5 |
| Type of surgery | THR | 3380 | 55.4 |
|  | TKR | 2717 | 44.6 |
| Revision surgery | < 2 hours or missing | 5922 | 97.1 |
|  | ≥ 2 hours | 175 | 2.9 |
| Geographic region | Western Europe | 2150 | 35.3 |
|  | Eastern Europe | 1263 | 20.7 |
|  | USA/Canada | 1188 | 19.5 |
|  | Other | 1496 | 24.5 |
| Sex | Female | 3695 | 60.6 |
|  | Male | 2402 | 39.4 |
| Body weight | < 70 kg | 2058 | 33.8 |
|  | 70–90 kg | 2642 | 43.3 |
|  | > 90 kg | 1397 | 22.9 |
| Low Hb prior to surgery (< 13 g/dL for men and < 12 g/dL for women) | No | 5070 | 83.2 |
|  | Yes | 1027 | 16.8 |
| Active malignancy | No | 6046 | 99.2 |
|  | Yes | 51 | 0.8 |
| Thrombophilia | No | 6091 | 99.9 |
|  | Yes | 6 | 0.1 |
| Severe varicosis | No | 5907 | 96.9 |
|  | Yes | 190 | 3.1 |
| History of bleeding | No | 5956 | 97.7 |
|  | Yes | 141 | 2.3 |
| History of hypertension | No | 2895 | 47.5 |
|  | Yes | 3202 | 52.5 |
| PAI or ASA use while receiving rivaroxaban | No | 5488 | 90.0 |
|  | Yes | 609 | 10.0 |
| NSAID use while receiving rivaroxaban | No | 1740 | 28.5 |
|  | Yes | 4357 | 71.5 |
| **Efficacy population (n = 4246)** | | | |
| Age | < 65 years | 2080 | 49.0 |
|  | 65–75 years | 1583 | 37.3 |
|  | > 75 years | 583 | 13.7 |
| Baseline renal function (CrCl) | > 80 mL/min | 2548 | 60.0 |
|  | 50–80 mL/min | 1438 | 33.9 |
|  | < 50 mL/min | 260 | 6.1 |
| Type of surgery | THR | 2458 | 57.9 |
|  | TKR | 1788 | 42.1 |
| Duration of surgery | < 2 hours or missing | 3333 | 78.5 |
|  | ≥ 2 hours | 913 | 21.5 |
| Geographic region | Western Europe | 1496 | 35.2 |
|  | Eastern Europe | 954 | 22.5 |
|  | USA/Canada | 808 | 19.0 |
|  | Other | 988 | 23.3 |
| Sex | Female | 2490 | 58.6 |
|  | Male | 1756 | 41.4 |
| Body weight | < 70 kg | 1438 | 33.9 |
|  | 70–90 kg | 1834 | 43.2 |
|  | > 90 kg | 974 | 22.9 |
| Body mass index | < 25 kg/m^2^ | 1053 | 24.8 |
|  | 25–<35 kg/m^2^ | 2753 | 64.8 |
|  | ≥ 35 kg/m^2^ | 440 | 10.4 |
| Any VTE risk factor | No | 3945 | 92.9 |
|  | Yes | 301 | 7.1 |
| History of VTE | No | 4147 | 97.7 |
|  | Yes | 99 | 2.3 |
| Cardiac insufficiency | No | 4186 | 98.6 |
|  | Yes | 60 | 1.4 |
| Active malignancy | No | 4210 | 99.2 |
|  | Yes | 36 | 0.8 |
| Thrombophilia | No | 4241 | 99.9 |
|  | Yes | 5 | 0.1 |
| Severe varicosis | No | 4117 | 97.0 |
|  | Yes | 129 | 3.0 |
| History of hypertension | No | 2071 | 48.8 |
|  | Yes | 2175 | 51.2 |
| History of CV disease | No | 2056 | 48.4 |
|  | Yes | 2190 | 51.6 |

*ASA* acetylsalicylic acid, *CrCl* creatinine clearance, *CV* cardiovascular, *Hb* hemoglobin, *NSAID* non-steroidal anti-inflammatory drug, *PAI* platelet aggregation inhibitor, *THR* total hip replacement, *TKR* total knee replacement, *VTE* venous thromboembolism

^a^Percentages are subject to rounding and may not add up to 100%

**Supplemental Table 3** Model-predicted rivaroxaban exposure summary

| **Exposure measure** | **P05** | **Median** | **P95** | **Mean** | **CV, %** |
| --- | --- | --- | --- | --- | --- |
| **Safety population (n = 6097)** | | | | | |
| C_trough_ (μg/L) | 3.10 | 7.03 | 17.1 | 8.21 | 66.6 |
| C_max_ (μg/L) | 106 | 127 | 163 | 130 | 14.2 |
| AUC_0–24_ (μg/L×h) | 941 | 1,211 | 1,767 | 1,265 | 22.4 |
| **Efficacy population (n = 4246)** | | | | | |
| C_trough_ (μg/L) | 3.10 | 7.07 | 16.7 | 8.13 | 61.3 |
| C_max_ (μg/L) | 105 | 127 | 162 | 129 | 14.0 |
| AUC_0–24_ (μg/L×h) | 939 | 1,206 | 1,759 | 1,258 | 21.7 |

*AUC_0–24_* area under the plasma concentration–time curve from 0 to 24 hours, *C_max_* maximum plasma concentration, *C_trough_* trough plasma concentration, *CV* coefficient of variation, *P05* 5th percentile, *P95* 95th percentile

**Supplemental Table 4** Observed event rates

|  | **Patients with event/total patients (%)** | |
| --- | --- | --- |
|  | **Days 1–4** | **After day 4** |
| **Efficacy outcome** | | |
| Total VTE | 180/4246 (4.2) | |
| **Safety outcomes** | | |
| 1. Major bleeding | 11/6097 (0.18) | 11/5995 (0.18) |
| 1. Major or NMCR bleeding | 82/6097 (1.3) | 91/5930 (1.5) |

*NMCR* non-major clinically relevant, *VTE* venous thromboembolism

**Supplemental Table 5** AIC values for safety and efficacy outcomes and exposure metrics resulting from univariate assessments. Selected exposure metrics (i.e., the metric with the lowest AIC value) for multivariate assessment are marked in bold

| **Outcome** | **AUC_0–24_** | **C_max_** | **C_trough_** |
| --- | --- | --- | --- |
| Total VTE | −7.15 | −**12.7** | −0.43 |
| Major bleeding (days 1–4) | −1.13 | −**6.43** | 1.90 |
| Major bleeding (after day 4) | −**0.0992** | 0.527 | −0.0580 |
| Major or NMCR bleeding (days 1–4) | 1.96 | −**0.426** | −0.417 |
| Major or NMCR bleeding (after day 4) | −19.1 | −8.55 | −**21.7** |

*AUC_0–24_* area under the plasma concentration–time curve from 0 to 24 hours, *AIC* Akaike information criterion, *C_max_* maximum plasma concentration, *C_trough_* trough plasma concentration, *NMCR* non-major clinically relevant, *VTE* venous thromboembolism

**Supplemental Table 6** Results of the full model for total VTE^a^

|  | **Log(OR) estimate** | **LLCI  (2.5%)** | **ULCI (97.5%)** | **p value** |
| --- | --- | --- | --- | --- |
| Exposure | | | | |
| C_max_ | –0.0053 | –0.0157 | 0.0043 | 0.2915 |
| Forced into ER | | | | |
| Age = 65–75 years | 0.3520 | –0.0463 | 0.7531 | 0.0833 |
| Age > 75 years | 0.8376 | 0.3373 | 1.3358 | 0.0011 |
| CrCl = 50–80 mL/min | 0.3891 | 0.0004 | 0.7793 | 0.0498 |
| CrCl < 50 mL/min | 0.2018 | –0.5163 | 0.8734 | 0.5712 |
| Potential covariates | | | | |
| Type of surgery = TKR | 1.6916 | 1.3042 | 2.1034 | 0.0000 |
| Region = other | 0.6449 | 0.2071 | 1.0863 | 0.0039 |
| Region = Eastern Europe | 0.1279 | –0.3488 | 0.5953 | 0.5953 |
| Region = USA/Canada | 0.0401 | –0.4290 | 0.5059 | 0.8661 |
| Revision surgery ≥ 2 hours | 0.3864 | 0.0288 | 0.7335 | 0.0345 |
| Sex = male | –0.3265 | –0.7219 | 0.0590 | 0.0975 |
| Body weight < 70 kg | –0.1414 | –0.5761 | 0.2840 | 0.5173 |
| Body weight > 90 kg | –0.0634 | –0.5714 | 0.4253 | 0.8022 |
| Body mass index < 25 kg/m^2^ | –0.3040 | –0.8078 | 0.1799 | 0.2210 |
| Body mass index ≥ 35 kg/m^2^ | –0.1245 | –0.7286 | 0.4529 | 0.6771 |
| Any VTE risk factor = yes | –0.6127 | –2.1810 | 1.0752 | 0.4612 |
| History of VTE = yes | 0.4188 | –1.0529 | 1.6513 | 0.5467 |
| Cardiac insufficiency = yes | 0.4529 | –1.6705 | 2.1444 | 0.6457 |
| Active malignancy = yes | 0.2859 | –2.2272 | 2.2429 | 0.7958 |
| Thrombophilia = yes | 2.5299 | –2.5064 | 5.2367 | 0.2297 |
| Severe varicosis = yes | 1.3948 | –0.2074 | 2.8585 | 0.0849 |
| History of hypertension = no | 0.0860 | –4.8830 | 2.7321 | 0.9576 |
| History of CV disease = no | –0.2703 | –2.9318 | 4.7039 | 0.8701 |

*C_max_* maximum plasma concentration, *CV* cardiovascular, *CrCl* creatinine clearance, *ER* exposure–response, *LLCI* lower limit of confidence interval, *OR* odds ratio, *TKR* total knee replacement, *ULCI* upper limit of confidence interval, *VTE* venous thromboembolism

^a^Results of the full model for total VTE are shown because exposure was not included in the final model for this outcome

**Supplemental Table 7** Results of the final model for total VTE

|  | **OR (95% CI)** | **p value** | **p value (LRT)** |
| --- | --- | --- | --- |
| Patient with vs. without TKR | 5.91 (4.09–8.76) | 0 | 0 |
| Age = 65–75 years (vs. < 65 years) | 1.38 (0.94–2.02) | 0.10 | 0.02 |
| Age > 75 years (vs. < 65 years) | 2.01 (1.25–3.20) | 0.004 |  |
| CrCl = 50–80 mL/min (vs. > 80 mL/min) | 1.46 (1.02–2.09) | 0.04 | 0.11 |
| CrCl < 50 mL/min (vs. > 80 mL/min) | 1.14 (0.59–2.10) | 0.68 |  |

*CI* confidence interval, *CrCl* creatinine clearance, *LRT* likelihood ratio test, *OR* odds ratio, *TKR* total knee replacement, *VTE* venous thromboembolism

**Supplemental Table 8** Results of the full exposure–safety models: outcomes for which exposure was excluded from the final models

|  | **Log(OR) estimate** | **LLCI  (2.5%)** | **ULCI (97.5%)** | **p value** |
| --- | --- | --- | --- | --- |
| **Major bleeding: days 1–4** | | | | |
| Exposure | | | | |
| C_max_ | –0.0706 | –0.1453 | –0.0061 | 0.0291 |
| Forced into ER | | | | |
| Age = 65–75 years | 1.0083 | –0.4685 | 2.4753 | 0.1753 |
| Age > 75 years | 2.1939 | 0.0067 | 4.2247 | 0.0494 |
| CrCl = 50–80 mL/min | –0.8124 | –2.7305 | 0.8695 | 0.3518 |
| CrCl < 50 mL/min | –0.7992 | –5.8613 | 2.1229 | 0.6244 |
| Potential covariates | | | | |
| Type of surgery = TKR | 1.3733 | 0.0074 | 2.9066 | 0.0487 |
| Region = other | –0.1790 | –2.0196 | 1.4461 | 0.8321 |
| Region = Eastern Europe | –0.5349 | –2.9061 | 1.2193 | 0.5715 |
| Region = USA/Canada | 0.2497 | –1.3183 | 1.8334 | 0.7511 |
| Revision surgery ≥ 2 hours | 1.6408 | –0.5989 | 3.1499 | 0.1258 |
| Sex = male | 1.4729 | –0.0661 | 3.3217 | 0.0613 |
| Body weight < 70 kg | 1.5499 | –0.1879 | 3.2861 | 0.0787 |
| Body weight > 90 kg | 0.2687 | –1.1436 | 1.7996 | 0.7105 |
| Low Hb prior to surgery (< 13 g/dL for men or  < 12 g/dL for women) = yes | 0.0847 | –2.1312 | 1.5657 | 0.9236 |
| Active malignancy = yes | 1.4156 | –3.5163 | 3.7353 | 0.4366 |
| Thrombophilia = yes | 3.1989 | –1.9196 | 6.1237 | 0.1591 |
| Severe varicosis = yes | 1.9045 | –0.4629 | 3.6025 | 0.1002 |
| History of bleeding = yes | 1.3312 | –0.9479 | 2.8764 | 0.2068 |
| History of hypertension = no | –0.3583 | –1.6439 | 0.8765 | 0.5680 |
| PAI or ASA use while receiving rivaroxaban = yes | –1.3308 | –6.2211 | 0.8448 | 0.2835 |
| NSAID use while receiving rivaroxaban = no | 0.2778 | –1.0222 | 1.4744 | 0.6579 |
| **Major bleeding: after day 4** | | | | |
| Exposure | | | | |
| AUC_0–24_ | 0.0007 | –0.0012 | 0.0019 | 0.3843 |
| Forced into ER | | | | |
| Age = 65–75 years | 0.3057 | –1.0798 | 1.7038 | 0.6591 |
| Age > 75 years | 0.5468 | –1.4323 | 2.3194 | 0.5644 |
| CrCl = 50–80 mL/min | –0.2269 | –1.7326 | 1.2285 | 0.7592 |
| CrCl < 50 mL/min | –1.0256 | –6.0279 | 1.6201 | 0.4931 |
| Potential covariates | | | | |
| Type of surgery = TKR | 0.8720 | –0.4553 | 2.3733 | 0.2017 |
| Region = other | –0.4256 | –2.2121 | 1.0790 | 0.5905 |
| Region = Eastern Europe | –1.6799 | –6.5603 | 0.5091 | 0.1555 |
| Region = USA/Canada | –0.3968 | –1.9142 | 1.0652 | 0.5933 |
| Revision surgery ≥ 2 hours | 0.5432 | –4.3197 | 2.6254 | 0.7301 |
| Sex = male | 0.3912 | –0.8439 | 1.6752 | 0.5327 |
| Body weight < 70 kg | –0.0838 | –1.9078 | 1.5239 | 0.9209 |
| Body weight > 90 kg | 0.1549 | –1.2659 | 1.5315 | 0.8246 |
| Low Hb prior to surgery (< 13 g/dL for men or < 12 g/dL for women) = yes | 0.3071 | –1.3551 | 1.5995 | 0.6794 |
| Active malignancy = yes | 0.9407 | –3.9246 | 3.0397 | 0.5721 |
| Thrombophilia = yes | 4.1916 | –0.8361 | 6.9217 | 0.0823 |
| Severe varicosis = yes | 0.3053 | –4.5730 | 2.4535 | 0.8429 |
| History of bleeding = yes | 1.2989 | –0.9495 | 2.8089 | 0.2101 |
| History of hypertension = no | –0.0480 | –1.3670 | 1.1610 | 0.9391 |
| PAI or ASA use while receiving rivaroxaban = yes | 1.4062 | 0.1081 | 2.5985 | 0.0350 |
| NSAID use while receiving rivaroxaban = no | –0.5136 | –2.1821 | 0.7811 | 0.4594 |
| **Major or NMCR bleeding: days 1–4** | | | | |
| Exposure | | | | |
| C_max_ | –0.0033 | –0.0202 | 0.0113 | 0.6731 |
| Forced into ER | | | | |
| Age = 65–75 years | 0.6460 | 0.1115 | 1.1864 | 0.0179 |
| Age > 75 years | 0.0952 | –0.8209 | 0.9420 | 0.8319 |
| CrCl = 50–80 mL/min | –0.1256 | –0.7142 | 0.4517 | 0.6714 |
| CrCl < 50 mL/min | 0.0946 | –1.1703 | 1.1702 | 0.8728 |
| Potential covariates | | | | |
| Type of surgery = TKR | 0.0677 | –0.4413 | 0.5719 | 0.7932 |
| Region = other | –0.7219 | –1.4925 | –0.0292 | 0.0408 |
| Region = Eastern Europe | –0.5091 | –1.2627 | 0.1628 | 0.1417 |
| Region = USA/Canada | 0.5223 | –0.0774 | 1.1164 | 0.0874 |
| Revision surgery ≥ 2 hours | 0.1822 | –1.3995 | 1.2653 | 0.7855 |
| Sex = male | 1.1817 | 0.6669 | 1.7158 | 0.0000 |
| Body weight < 70 kg | 0.3829 | –0.2378 | 0.9876 | 0.2232 |
| Body weight > 90 kg | 0.0759 | –0.4823 | 0.6267 | 0.7879 |
| Low Hb prior to surgery (< 13 g/dL for men or < 12 g/dL for women) = yes | –0.1419 | –0.8625 | 0.4789 | 0.6705 |
| Active malignancy = yes | 0.3585 | –1.8531 | 1.7369 | 0.6903 |
| Thrombophilia = yes | 1.7083 | –3.1813 | 3.9327 | 0.3559 |
| Severe varicosis = yes | 0.8442 | –0.2932 | 1.7299 | 0.1318 |
| History of bleeding = yes | 1.1551 | 0.2768 | 1.8948 | 0.0124 |
| History of hypertension = no | 0.0649 | –0.4057 | 0.5330 | 0.7859 |
| PAI or ASA use while receiving rivaroxaban = yes | –0.2894 | –1.0954 | 0.3899 | 0.4242 |
| NSAID use while receiving rivaroxaban = no | –0.1722 | –0.6900 | 0.3116 | 0.4934 |

*ASA* acetylsalicylic acid, *AUC_0–24_* area under the plasma concentration–time curve from 0 to 24 hours, *C_max_* maximum plasma concentration, *CrCl* creatinine clearance, *ER* exposure–response, *Hb* hemoglobin, *LLCI* lower limit of confidence interval, *NMCR* non-major clinically relevant, *OR* odds ratio, *NSAID* non-steroidal anti-inflammatory drug, *PAI* platelet aggregation inhibitor, *TKR* total knee replacement, *ULCI* upper limit of confidence interval

**Supplemental Table 9** Results of the final safety outcome models

|  | **OR (95% CI)** | **p value** | **p value (LRT)** |
| --- | --- | --- | --- |
| **Major bleeding: days 1–4** | | | |
| Age = 65–75 years (vs. < 65 years) | 1.61 (0.42–5.79) | 0.47 | 0.30 |
| Age > 75 years (vs. < 65 years) | 4.41 (0.64–21.84) | 0.12 |  |
| CrCl = 50–80 mL/min (vs. > 80 mL/min) | 0.27 (0.04–1.18) | 0.08 | 0.18 |
| CrCl < 50 mL/min (vs. > 80 mL/min) | 0.19 (0.001–2.24) | 0.22 |  |
| **Major bleeding: after day 4** | | | |
| Age = 65–75 years (vs. < 65 years) | 1.89 (0.49–7.44) | 0.35 | 0.45 |
| Age > 75 years (vs. < 65 years) | 3.10 (0.43–17.62) | 0.24 |  |
| CrCl = 50–80 mL/min (vs. > 80 mL/min) | 0.69 (0.17–2.64) | 0.59 | 0.69 |
| CrCl < 50 mL/min (vs. > 80 mL/min) | 0.32 (0.002–3.67) | 0.41 |  |
| **Major or NMCR bleeding: days 1–4** | | | |
| Region = other (vs. Western Europe) | 0.48 (0.23–0.94) | 0.03 | 0.001 |
| Region = Eastern Europe (vs. Western Europe) | 0.58 (0.27–1.13) | 0.11 |  |
| Region = USA/Canada  (vs. Western Europe) | 1.63 (0.98–2.70) | 0.06 |  |
| Sex = male (vs. female) | 3.05 (1.92–4.93) | < 0.00001 | < 0.00001 |
| Age = 65–75 years (vs. age < 65 years) | 1.76 (1.07–2.93) | 0.03 | 0.05 |
| Age > 75 years (vs. age < 65 years) | 1.02 (0.42–2.27) | 0.97 |  |
| CrCl = 50–80 mL/min (vs. > 80 mL/min) | 0.93 (0.54–1.57) | 0.78 | 0.92 |
| CrCl < 50 mL/min (vs. > 80 mL/min) | 1.12 (0.34–2.97) | 0.84 |  |
| **Major or NMCR bleeding: after day 4** | | | |
| C_trough_ | 1.05 (1.03–1.07) | < 0.00001 | < 0.00001 |
| Age = 65–75 years (vs. < 65 years) | 1.13 (0.68–1.88) | 0.63 | 0.48 |
| Age > 75 years (vs. < 65 years) | 1.52 (0.76–2.96) | 0.23 |  |
| CrCl = 50–80 mL/min (vs. > 80 mL/min) | 0.88 (0.53–1.46) | 0.63 | 0.87 |
| CrCl < 50 mL/min (vs. > 80 mL/min) | 0.84 (0.34–1.87) | 0.68 |  |

*CI* confidence interval, *CrCl* creatinine clearance, *C_trough_* trough plasma concentration, *LRT* likelihood ratio test, *NMCR* non-major clinically relevant, *OR* odds ratio
